# Improving Clerkship to Enhance Patients’ Quality of care (ICEPACQ): a baseline study

**DOI:** 10.1186/s12913-024-11337-w

**Published:** 2024-07-26

**Authors:** Kennedy Pangholi, Enid Kawala Kagoya, Allan G Nsubuga, Irene Atuhairwe, Prossy Nakattudde, Brian Agaba, Bonaventure Ahaisibwe, Esther Ijangolet, Eric Otim, Paul Waako, Julius Wandabwa, Milton Musaba, Antonina Webombesa, Kenneth Mugabe, Ashley Nakawuki, Richard Mugahi, Faith Nyangoma, Jesca Atugonza, Elizabeth Ajalo, Alice Kalenda, Ambrose Okibure, Andrew Kagwa, Ronald Kibuuka, Betty Nakawuka, Francis Okello, Proscovia Auma

**Affiliations:** 1 Faculty of Health Science, Busitema University, P.O. Box 1460, Mbale, Uganda; 2 Institute of Public Health Department of Community Health, Busitema University, faculty if Health Sciences, P.O. Box 1460, Mbale, Uganda; 3 Seed Global Health, P.O. Box 124991, Kampala, Uganda; 4 Department of Pharmacology and Therapeutics, Busitema University, Faculty of Health Science, P.O. Box 1460, Mbale, Uganda; 5 Department of Obstetrics and Gynecology, Busitema University, Faculty of Health Sciences, P.O. Box 1460, Mbale, Uganda; 6 Department of Obstetrics and Gynecology, Mbale Regional Referral Hospital, P.O. Box 921, Mbale, Uganda; 7 Department of Nursing, Busitema University, Faculty of Health Sciences, P.O. Box 1460, Mbale, Uganda; 8 Ministry of Health, Plot 6, Lourdel Road, Nakasero, P.O. Box 7272, Kampala, Uganda

**Keywords:** Clerkship, Gynecology ward

## Abstract

**Background:**

Proper and complete clerkships for patients have long been shown to contribute to correct diagnosis and improved patient care. All sections for clerkship must be carefully and fully completed to guide the diagnosis and the plan of management; moreover, one section guides the next. Failure to perform a complete clerkship has been shown to lead to misdiagnosis due to its unpleasant outcomes, such as delayed recovery, prolonged inpatient stay, high cost of care and, at worst, death.

**Objective:**

The objectives of the study were to determine the gap in clerkship, the impact of incomplete clerkship on the length of hospital stay, to explore the causes of the gap in clerkship of the patients and the strategies which can be used to improve clerkship of the patients admitted to, treated and discharged from the gynecological ward in Mbale RRH.

**Methodology:**

This was a mixed methods study involving the collection of secondary data via the review of patients’ files and the collection of qualitative data via key informant interviews. The files of patients who were admitted from August 2022 to December 2022, treated and discharged were reviewed using a data extraction tool. The descriptive statistics of the data were analyzed using STATA version 15, while the qualitative data were analyzed via deductive thematic analysis using Atlas ti version 9.

**Results:**

Data were collected from 612 patient files. For qualitative data, a total of 8 key informant interviews were conducted. Social history had the most participants with no information provided at all (83.5% not recorded), with biodata and vital sign examination (20% not recorded) having the least number. For the patients’ biodata, at least one parameter was recorded in all the patients, with the greatest gap noted in terms of recording the nearest health facility of the patient (91% not recorded). In the history, the greatest gap was noted in the history of current pregnancy (37.5% not provided at all); however, there was also a large gap in the past gynecological history (71% not recorded at all), past medical history (71% not recorded at all), past surgical history (73% not recorded at all) and family history (80% not recorded at all). The physical examination revealed the greatest gap in the abdominal examination (43%), with substantial gaps in the general examination (38.5% not recorded at all) and vaginal examination (40.5% not recorded at all), and the vital sign examination revealed the least gap. There was no patient who received a complete clerkship. There was a significant association between clerkships and the length of hospital stay. The causes of the gap in clerkships were multifactorial and included those related to the hospital, those related to the health worker, those related to the health care system and those related to the patient. The strategies to improve the clerkship of patients also included measures taken by health care workers, measures taken by hospitals and measures taken by the government.

**Conclusion and recommendation:**

There is a gap in the clerkships of patients at the gynecological ward that is recognized by the stakeholders at the ward, with some components of the clerkship being better recorded than others, and no patients who received a complete clerkship. There was a significant association between clerkships and the length of hospital stay.

The following is the recommended provision of clerkship tools, such as the standardized clerkship guide and equipment for patient examination, continuous education of health workers on clerkships and training them on how to use the available tools, the development of SOPs for patient clerkships, the promotion of clerkship culture and the supervision of health workers.

**Supplementary Information:**

The online version contains supplementary material available at 10.1186/s12913-024-11337-w.

## Introduction

A complete clerkship is the core upon which a medical diagnosis is made, and this depends on the patient’s medical history, the signs noticed on physical examination, and the results of laboratory investigations [1]. These sections of the clerkship should be completed carefully and appropriately to obtain a correct diagnosis; moreover, one part guides the next. A complete gynecological clerkship comprises the patient’s biodata, presenting complaint, history of presenting complaint, review of systems, past gynecological history, past obstetric history, past medical history, past surgical history, family history, social history, physical examination, laboratory investigation, diagnosis and management plan [2, 3].

History taking, also known as medical interviews, is a brief personal inquiry and interrogation about bodily complaints by the doctor to the patient in addition to personal and social information about the patient [4]. It is estimated that 70-90% of a medical diagnosis can be determined by history alone [5, 6]. Physical examination, in addition to the patient’s history, is equally important because it helps to discover more objective aspects of the disease [7]. The investigation of the patient should be guided by the findings that have been obtained on history taking and the physical examination [1].

Failure to establish a good complete and appropriate clerkship for patients leads to diagnostic uncertainties, which are associated with unfavorable outcomes. Some of the effects of poor clerkship include delayed diagnosis and inappropriate investigations, which lead to unnecessary expenditures on irrelevant tests and drugs and other effects, such as delayed recovery, prolonged inpatient stays, high costs of care and, at worst, death [8, 9]. Despite health care workers receiving training in medical school about the relevance of physical examination, this has been poorly practiced and replaced with advanced imaging techniques such as ultrasounds, CT scans, and MRIs, which continue to make health care services unaffordable for most populations in developing countries [6]. In a study conducted to determine the prevalence and classification of misdiagnosis among hospitalized patients in five general hospitals in central Uganda, 9.2% of inpatients were misdiagnosed, and these were linked to inadequate medical history and examination, as the most common conditions were the most commonly misdiagnosed [9].

At Mbale RRH, there has been a progressive increase in the number of patients included in the gynecology department, which is expected to have compromised the quality of the clerkships that patients receive at the hospital [10]. However, there is limited information about the quality and completeness of clerkships for patients admitted to and treated at Mbale RRH. The current study therefore aimed to determine the gap in patient clerkships and the possible causes of these gaps and to suggest strategies for improving clerkships.

## Methods and materials

### Study design

This was a baseline study, which was part of a quality improvement project aimed at improving the clerkships of patients admitted and treated at Mbale RRH. This mixed cross-sectional survey employing both quantitative and qualitative techniques was carried out from August 2022 to December 2022. Both techniques were employed to triangulate the results and address the gap in clerkship using quantitative techniques. Then, qualitative methods were used to explain the reasons for the observed discrepancy, and strategies to improve clerkship were suggested.

### Study setting

The study was carried out in Mbale RRH, at the gynecologic ward. The hospital is in Mbale Municipal Council, 214 km to the east of the capital city of Kampala. It is the main regional referral hospital in the Elgon zone in eastern Uganda, a geographic area that borders the western part of Kenya. The Mbale RRH serves a catchment population of approximately 5 million people from 16 administrative districts. It is the referral hospital for the districts of Busia, Budaka, Kibuku, Kapchorwa, Bukwo, Butaleja, Manafwa, Mbale, Pallisa, Sironko and Tororo. The hospital is situated at an altitude of 1140 m within a range of 980–1800 m above sea level. Over 70% of inhabitants in this area are of Bantu ethnicity, and the great majority are part of rural agrarian communities. The Mbale RRH is a government-run, not-for-profit and charge-free 470-bed capacity that includes four major medical specialties: Obstetrics and Gynecology, Surgery, Internal Medicine, and Pediatrics and Child Health.

### Study population, sample size and sampling strategy

We collected the files of patients who were admitted to the gynecology ward at Mbale RRH from August 2022 to December 2022. All the files were selected for review. We also interviewed health workers involved in patient clerkships at the gynecological ward. For qualitative data, participants were recruited until data saturation was reached.

### Data collection

We collected both secondary and primary data. Secondary data were collected by reviewing the patients’ files. We identified research assistants who were trained in the data entry process. The data collection tool on Google Forms was distributed to the gadgets that were given to the assistants to enter the data. The qualitative data collection was performed via key informant interviews of the health workers involved in the clerkship of the patients, and the interviews were performed by the investigators. The selection of the participants was purposive, as we opted for those who clerk patients. After providing informed consent, the interview proceeded, with a voice recorder used to capture the data collected during the interview process and brief key notes made by the interviewer.

### Data collection tool

A data abstraction tool was developed and fed into Google Forms, which were used to collect information about patients’ clerkships from patients’ files. The tool was developed by the investigators based on the requirements of a full clerkship, and it acted as a checklist for the parameters of clerkships that were provided or not provided. The validity of this tool was first determined by using it to collect information from ten patients’ files, which were not included in the study, and the tool was adjusted accordingly. The tool for collecting the qualitative information was an interview guide that was developed by the interviewer and was piloted with two health workers. Then, the guide was adjusted before it was used for data collection.

### Variable handling

The dependent variable in the current study was the length of hospital stay. This was calculated from the date of admission and the date of discharge. There were two outcomes: “prolonged hospital stay” and “not prolonged”. A prolonged hospital stay was defined as a hospital stay of more than the 75^th^ percentile, according to a study conducted in Ethiopia [9]. This duration was more than 5 (five) days in the current study. The independent variables were the components of the clerkship.

### Data analysis

Data analysis was performed using STATA version 15. Univariate, bivariate and multivariate analyses were performed. Continuous variables were summarized using measures of central tendency and measures of dispersion, while categorical variables were summarized using frequencies and proportions. Bivariate analysis was performed using chi-square or Fischer’s exact tests, one-way ANOVA and independent t tests, with the level of significance determined by a p value of <= 0.2. Multivariate analysis was performed using logistic regression, and the level of significance was determined by a p value of <=0.05.

Qualitative data were analyzed using Atlas Ti version 9 via deductive thematic analysis. The audio recordings were transcribed, and the transcripts were then imported into Atlas Ti.

## Results

### Qualitative

The files of a total of 612 patients were reviewed.

### The gap in the clerkships of patients

#### Patient biodata

**Fig. 1:**
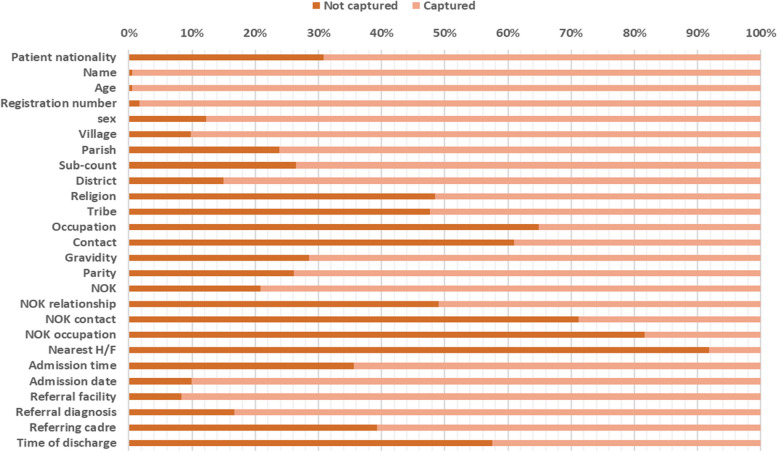
The gap in patients’ biodata

As shown in Fig. 1 below, at least one parameter under patient biodata was recorded for all the patients. The largest gap was identified in the recording of the nearest health facility of the patient, where 91% of the patients did not have this recorded, and the smallest gap was in the recording of the name and age, where less than 1% had this not recorded.

#### Compliance, HPC and ROS

**Fig. 2:**
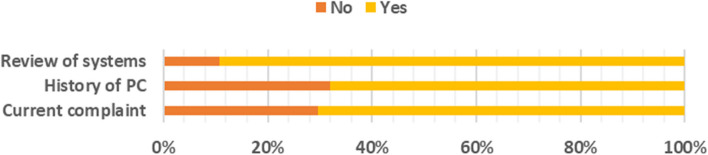
Gap in the presenting of complaints, HPCs and ROS

As shown in Fig. 2 below, the largest gap here was in recording the history of presenting complaint, which was not recorded in 32% of the participants. The least gap was in the review of systems, where it was not recorded in only 10% of the patients.

#### History

**Fig. 3:**
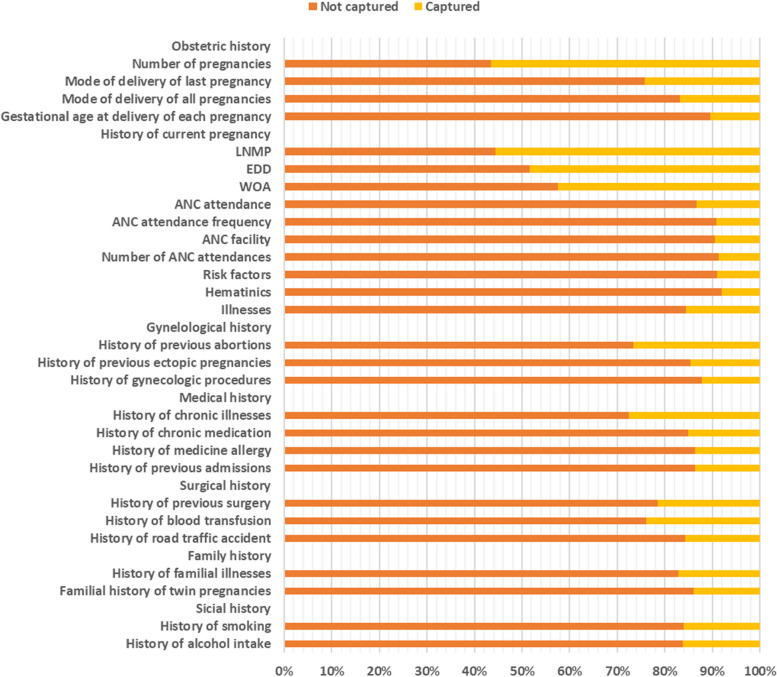
Gap in history

As shown in Fig. 3 below, the past obstetric history had the greatest gap in recording the gestational age at delivery of each pregnancy (89% not recorded), while the least gap was in recording the number of pregnancies (43% not recorded). In terms of the history of current pregnancy, the greatest gap was in recording whether hematinics were given to the mother (92% not recorded), while the least gap was in recording the date of the first day of the last normal menstrual period (LNMP) (44% not recorded). On other gynecological history, the largest gap was in recording the history of gynecological procedures (88% not recorded), while the least gap was in the history of abortions (73% not recorded). In the past medical history, the largest gap was in terms of history of medication allergies and history of previous admissions (86% not recorded), and the smallest gap was in terms of history of chronic illnesses (72% not recorded). In the past surgical history, the largest gap was in the history of trauma (84% not recorded), while the least gap was in the history of blood transfusion (76% not recorded). In terms of family history, there was a greater gap in the family history of twin pregnancies (86% not recorded) than in the family history of familial illnesses (83% not recorded). In terms of social history, neither alcohol intake nor smoking were recorded for 84% of the patients.

#### Physical examination

**Fig. 4:**
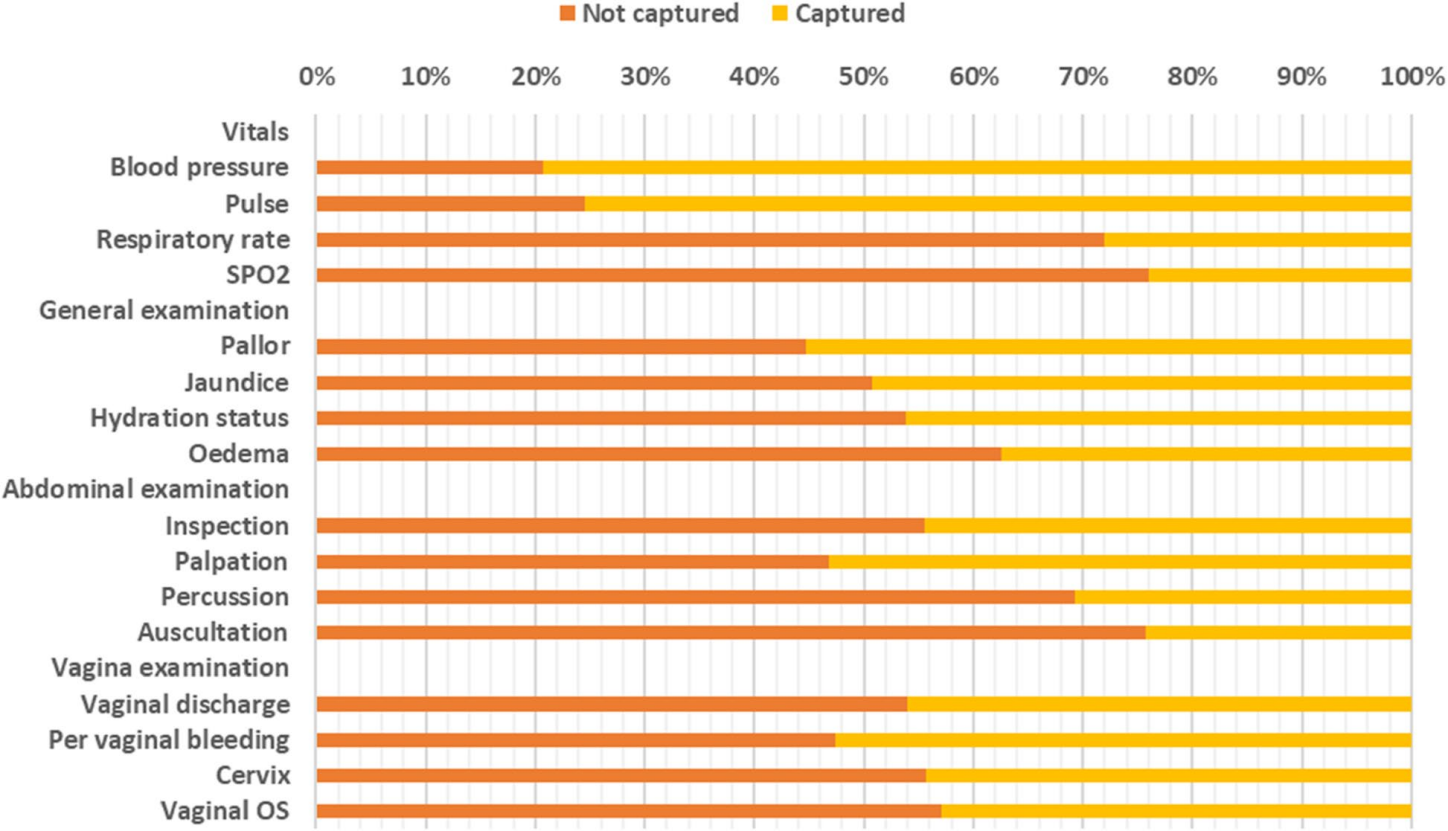
Gap in physical examination

As shown in Fig. 4 below, the least recorded vital sign was oxygen saturation (SPO2), with 76% of the patients’ SPO2 not being recorded, while blood pressure was least recorded (21% not recorded). On the general examination, checking for edema had the greatest gap (63% not recorded), while checking for pallor had the least gap (45% not recorded). On abdominal examination, auscultation had the greatest gap (76% not recorded), while inspection of the abdomen had the least gap (56% not recorded). On vaginal examination, the greatest difference was in examining the vaginal OS (57% not recorded), while the least difference was in checking for vaginal bleeding (47% not recorded).

#### Investigations, provisional diagnosis and management plan

**Fig. 5:**
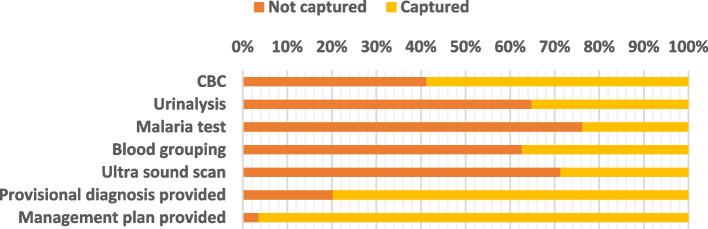
Gap in the provisional diagnosis and management plan

As shown in Fig. 5 below, the least common investigation was the malaria test (76% not performed), while the most common investigation was the CBC test (41% not performed). Provisional diagnosis was not performed in 20% of the patients. A management plan was not provided for approximately 4-5 of the patients.

#### Summary of the gap in clerkships

**Fig. 6:**
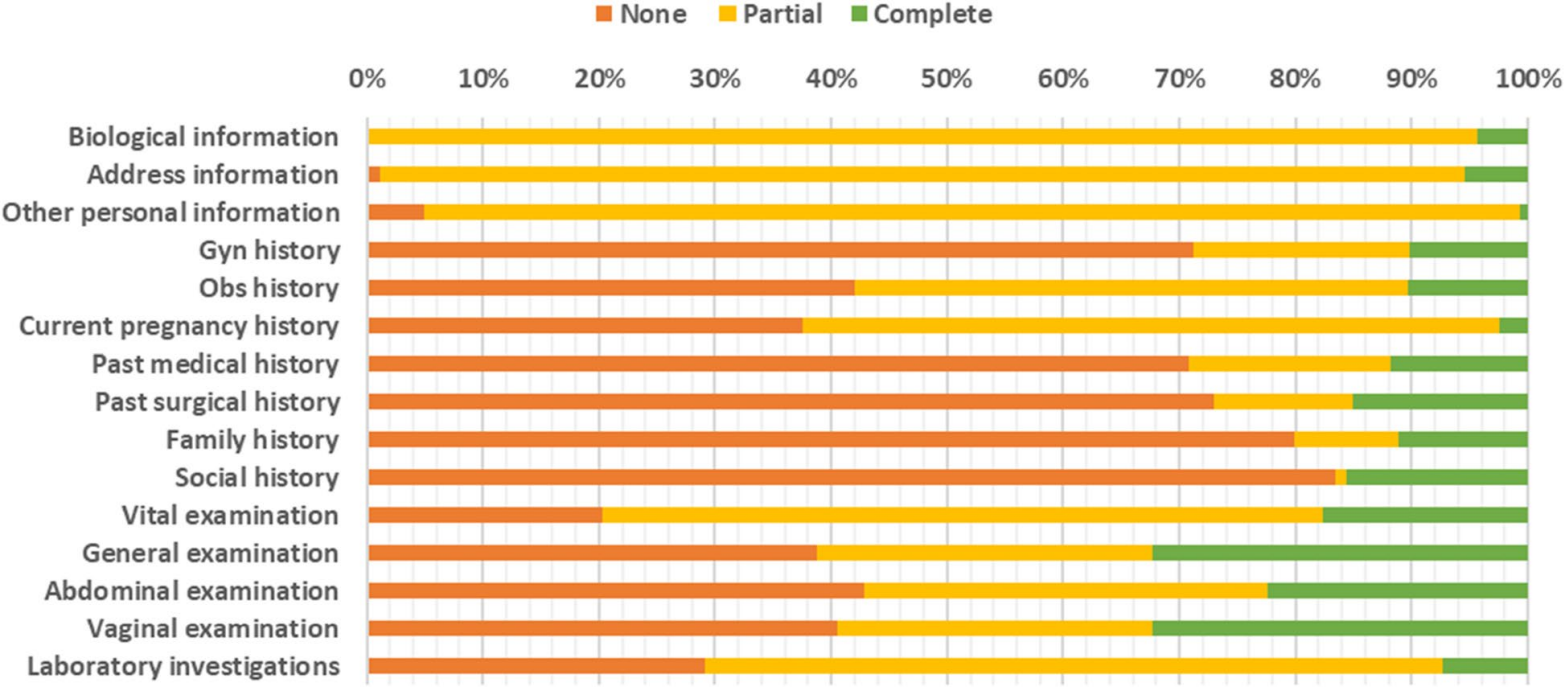
Summary of the gaps in clerkships

As shown in Fig. 6 below, most participants had a social history with no information provided at all, while biodata and vital sign examinations had the least number of participants with no information provided at all. There was no patient who had a complete clerkship.

#### Days of hospitalization

**Fig. 7:**
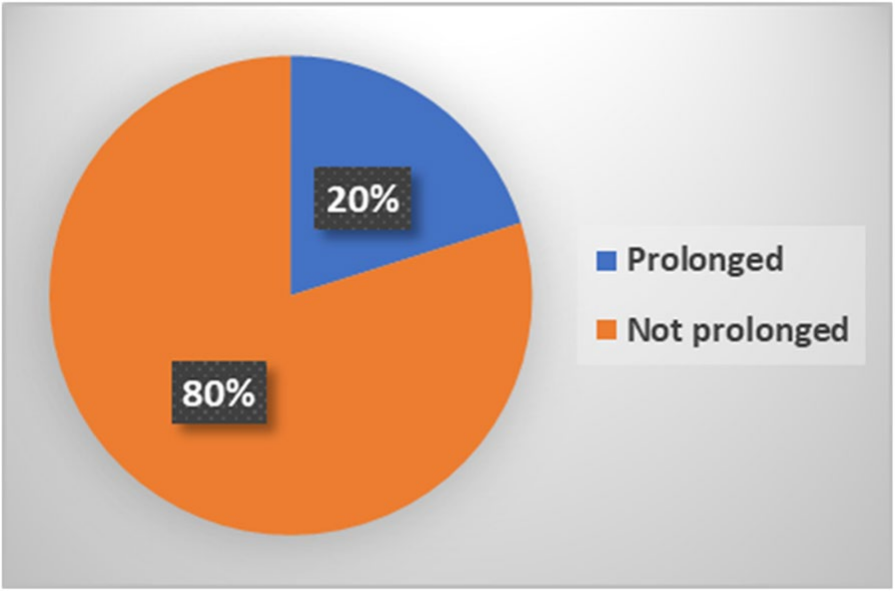
Duration of hospitalization

The days of hospitalization were not normally distributed and were positively skewed, with a median of 3 [2, 5] days. The mean days of hospitalization was 6.2 (±11.1). As shown in Fig. 7 below, 20% of the patients had prolonged hospitalization.

#### The effect of the clerkship gap on the number of days of hospital stay

As shown in Tables 1 and 2 below, the clerkship components that had a significant association with the days of hospitalization at the bivariate level included vital examination, abdominal examination, history of presenting complaint and treatment plan.

**Table 1:** Bivariate analysis showing the effect of the clerkship gap on the number of days of hospital stay

**Variable**	**Outcomes**	**Days of hospitalization**		**Chi**^2^*p* value
		**Not prolonged**	**Prolonged**	
Biological information	None	-	-	
	Partial	462(94.5)	118(95.9)	0.517
	Complete	27(5.5)	5(4.1)	
Address information	None	7(1.4)	0	
	Partial	249(50.9)	68(55.3)	0.316
	Complete	233(47.7)	55(44.7)	
Other personal information	None	4(0.8)	0	
	Partial	282(98.6)	123(100)	0.410
	Complete	3(0.6)	0	
Gyn history	None	347(71)	89(72.4)	
	Partial	90(18.4)	24(19.5)	0.705
	Complete	52(10.6)	10(8.1)	
Obs history	None	205(41.9)	52(42.3)	
	Partial	232(47.4)	60(48.8)	0.855
	Complete	52(10.6)	11(8.9)	
Current pregnancy history	None	181(37)	49(39.8)	
	Partial	296(60.5)	71(57.7)	0.845
	Complete	12(2.5)	3(2.4)	
Past medical history	None	349(71.4)	84(68.3)	
	Partial	85(17.4)	22(17.9)	0.705
	Complete	55(11.3)	17(13.8)	
Past surgical history	None	358(73.2)	89(72.4)	
	Partial	57(11.7)	16(13)	0.915
	Complete	74(15.1)	18(14.6)	
Family history	None	393(80.4)	96(78.1)	
	Partial	41(8.4)	14(11.4)	0.580
	Complete	55(11.3)	13(10.6)	
Social history	None	409(83.6)	102(82.9)	
	Partial	4(0.8)	1(0.8)	0.981
	Complete	76(15.4)	20(16.3)	
Vital examination	**None**	**107(21.9)**	**17(13.8)**	
	**Partial**	**299(61.2)**	**81(65.9)**	0.127*
	**Complete**	**83(17)**	**25(20.3)**	
General examination	None	193(39.5)	44(35.8)	
	Partial	136(27.8)	41(33.3)	0.476
	Complete	160(32.7)	38(30.9)	
Abdominal examination	**None**	**228(46.6)**	**34(27.6)**	
	**Partial**	**150(30.7)**	**63(51.2)**	0.001^*^
	**Complete**	**111(22.7)**	**26(21.1)**	
Vaginal examination	None	201(41.1)	47(38.2)	
	Partial	132(27)	34(27.6)	0.831
	Complete	156(31.9)	42(34.2)	
Laboratory investigations	None	143(29.2)	35(28.5)	
	Partial	310(63.4)	79(64.2)	0.984
	Complete	36(7.4)	9(7.3)

**Table 2:** Bivariate analysis showing the effect of the clerkship gap on the number of days of hospital stay

**Variable**	**Outcomes**	**Days of hospitalization**		**Chi**^**2**^ ***p*** **value**
		**Not prolonged**	**Prolonged**	
Presenting complaint	Not Provided	150(30.7)	32(26)	0.312
	Provided	339(69.3)	91(73.4)	
History of presenting complaint	**Not provided**	**165(33.7)**	**31(25.2)**	0.070*
	**Provided**	**324(66.3)**	**92(74.8)**	
Review of systems	Not provided	275(56.2)	62(50.4)	0.245
	Provided	214(43.8)	61(49.6)	
Management plan	Not provided	18(3.7)	4(3.3)	0.819
	Provided	471(96.3)	119(96.8)	
Treatment plan	**Not provided**	**37(7.6)**	**3(2.4)**	0.04*
	**Provided**	**452(92.4)**	**120(97.6)**	

As shown in Table 3, the only clerkship component that had a significant association with the days of hospitalization at the multivariate level was abdominal examination. People who had partial abdominal examinations were 1.9 times more likely to have prolonged hospital stays than those who had complete abdominal examinations.

**Table 3:** The effect of the clerkship gap on the number of days of hospital stay according to multivariate analysis

**Variable**	**Outcomes**	**Adjusted odds ratio**	**95%CI**	**p value**
Vital examination	None	Reference		
	Partial	1.4	0.7-2.6	0.236
	Complete	1.7	0.2-3.4	0.160
Abdominal examination	None	0.7	0.4-1.3	0.335
	**Partial**	**1.9**	**1.1-3.2**	0.020*
	Complete	Reference		
History of presenting complaint	Not provided	Reference		
	Provided	1.3	0.8-2.1	0.219
Treatment plan	Not provided	Reference		
	Provided	2.5	0.7-7.4	0.142

#### Qualitative results

We conducted a total of 8 key informant interviews with the following characteristics as shown in table 4 below.

**Table 4:** The characteristics of key informants

Participant ID	Age	Sex	Cadre	Work experience in years
KII1	42	Male	Medical officer special grade, obstetrics, and gynecology	18
KII2	30	Female	Medical officer	6
KII3	35	Male	Medical intern/Clinical officer	15
KII4	54	Female	Diploma midwife	30
KII5	53	Female	Diploma midwife	30
KII6	30	Female	Medical officer	5
KII7	45	Female	Intern midwife/Diploma nurse	29
KII8	53	Female	Bachelor midwife	27

The qualitative results are summarized in Table 5 below.

**Table 5:** Summary of the qualitative results

**Theme**	**Subtheme**	**Code**
State of the clerkship		• Quality of clerkship is not good, it is lacking, incomplete most of the times • However, it depends on several factors; who is clerking, how sick the patient is, the number of patients to be seen that particular day and the number of hours a person clerking has thus far worked
Who usually sees the patients		• Midwives, medical students, medical officers, specialists, Junior house officers
Challenges affecting clerkship	Health worker related	• Poor attitude of the health workers • Knowledge gap among health workers about clerkship • Lack of confidence by some health workers and students to commit to a diagnosis and management plan • Failure of laboratory personnel to do required investigations on time • Forgetfulness of some components of clerkship • Some health workers find taking notes tedious work
	Hospital related	• Lack of clerkship tools • Poor patient triaging • Poor clerkship tools available • Relativeness due to absence of supervision and emphasis on good clerkship • Clerkship done by wrong people who cannot do it the right way • Absence of specialized Doctors in the OPD department
	Health system related	• Understaffing • High patient load • Defective referral system, leading to overcrowding of hospital
	Patient related	• Emergency nature of some cases • Poverty of patients makes them unable to afford books and files
Strategies to improve clerkship	Measures to be taken by health worker	• Mentorship of students by the seniors on clerkship • Holding each other accountable as regards to the completeness of clerkship. • Providing feedback to each other and from the records department • Students should clerk and present to the seniors for diagnosis and management plan • Change of attitude of health workers, including laboratory personnel • The right people should clerk the patients
	Measures to be taken by hospital leadership	• Continuous education of clinicians on clerkship • Providing clerkship tools and making their availability sustainable like the logistics and equipment for patient examination • Develop a reward system to reward the health workers that do complete patient clerkship • Develop SOPs that require patients to have full clerkship at different stages, e.g., before entering theater • Provide printer for making the tools • Improve clerkship culture and make it a norm • Provide a good conducive room for patient examination. • Proper keeping of records so that information can easily be retrieved • Supervision of the staff • Provide electronic clerkship tools like computers • Have a records assistant to take the notes
	Measures to be taken by government	• Improve staffing • Streamline the referral system • Good motivation of staff • Equip the lower health facilities • Providing refresher courses about clerkship to the health workers

### The quality of clerkships on wards

It was reported that both the quality and completeness of clerkships on the ward are poor.

*“…most are not clerking fully the patients, just put in like biodata three items name, age address, then they go on the present complaint, diagnosis then treatment; patient clerkship is missing out some important information…” (KIISAMW 2)*

It was, however, noted that the quality of a clerkship depends on several factors, such as who is clerking, how sick the patient is, the number of patients to be seen that particular day and the number of hours a person clerks.

*“…so, the quality of clerkship is dependent on who is clerking but also how sick the patient is…” (KIIMO 3)*

### Which people usually clerk patients on the ward?

The following people were identified as those who clerking patients, midwives, medical students, junior house officers, medical officers and specialists.

*“…everyone clerks patients here; nurses, midwives, doctors, medical students, specialists, everyone as long as you are a health care provider…” (KIIMO 2)*

### Causes of the gaps in clerkships

These factors were divided into factors related to health workers, hospital-related factors, health system-related factors and patient-related factors.

#### Hospital-related factors

The absence of clerkship tools such as a standardized clerkship guide and equipment for the examination of patients, such as blood pressure machines, thermometers, and glucometers, among others, were among the reasons for the poor clerkships of the patients.

*…of course, there are other things like BP machines, thermometers; sometimes you want to examine a patient, but you don’t have those examining tools…” (KIIMO 1)*

The tools that were available were plain, and they play little role in facilitating clerkships. They reported that they end up using small exercise books with no guidance for easy clerkship and with limited space.

*“…most of our tools have these questions that are open ended and not so direct, so the person who is not so knowledgeable in looking out for certain things may miss out on certain data…” (KIIOG 1)*

The reluctance of some health workers to clerk patients fully was also reported to be because it is the new normal, and everyone follows a bandwagon to collect only limited information from patients because there is no one to follow up or supervise.

*“…you know when you go to a place, what you find people doing is what you also end up doing; I think it is because of what people are doing and no one is being held accountable for poor clerkship…” (KIIMO 3)*

The absence of specialized doctors in the OPD department forces most patients, even stable patients, to be managed by the OPD to crowd the ward, making complete clerkships for all patients difficult. Poor triaging of the patients was also noted as one of the causes of poor clerkship, as emergency cases are mixed with stable cases.

*“…and this gyn ward is supposed to see emergency gynecological cases, but you find even cases which are supposed to be in the gyn clinic are also here; so, it creates large numbers of people who need services…” (KIIMO 1)*

Clerkships being performed by the wrong people were also noted. It was emphasized that it is only a medical doctor who can perform good clerkships for patients, and any other cadres who perform clerkships contribute to poor clerkships on the ward.

#### Health worker-related factors

A poor attitude of health workers was reported, and it was found that many health workers consider complete clerkship to be a practice that is performed by people who do not know what they look for to make a diagnosis.

A lack of knowledge about clerkships is another factor that has been reported. Some health workers were reported to forget some of the components of clerkship; hence, they end up recording only what they remember at the time of clerkship.

A lack of confidence by some health workers and students that creates fear of committing to making a diagnosis and drawing a management plan was reported to hinder some of them from doing a complete clerkship of the patients.

*“…a nurse or a student may clerk, but they don’t know the diagnosis; so, they don’t want to commit themselves to a diagnosis…” (KIIMO 2)*

Some health workers reported finding the process of taking notes while clerking tedious; hence, they collected only limited information that they could write within a short period of time.

#### Health system-related factors

Understaffing of the ward was noted to cause a low health worker-to-patient ratio. This overworked the health workers due to the large numbers of patients to be seen.

*“…due to the thin human resource for health, many patients have to be seen by the same health worker, and it becomes difficult for one to clerk adequately; they tend to look out for key things majorly…” (KIIOG 1)*

It was noted that in the morning or at the start of a shift, the clerkship can be fair, but as the day progresses, the quality of the clerkship decreases due to exhaustion.

*“…you can’t clerk the person you are seeing at 5 pm the same way you clerked the person you saw at 9 am…” (KIIMO 3)*

The large numbers of patients were also associated with other factors, such as the inefficient referral system, where patients who can be managed in lower health facilities are also referred to Mbale RRH. It was also stated that some patients do not understand the referral system, causing self-referral to the RRH. Other factors that contributed to the poor referral system were limited trust of the patients, drug stockouts, limited skilled number of health workers, and limited laboratory facilities in the lower health facilities.

*“…so, everyone comes in from wherever they can, even unnecessary referrals from those lower health facilities make the numbers very high…” (KIIMO 1)*

#### Patient-related factors

It was reported that the nature of some cases does not allow the health worker to collect all the information from such a patient, for example, the emergency cases. However, some responders stated the emergent nature of the cases to be a contributor to the complete clerkship of such a patient, as the person clerking such a case is more likely to call for help, so they must have enough information on the patient. Additionally, they do not want to fill the gap in the care of this critical patient.

*“…usually, a more critical patient gets a more elaborate clerkship compared to a more stable one, where we will get something quick…” (KIIMO 3)*

The poor health of some patients makes them unable to afford the files and books where clerkship notes are to be taken.

*“…a patient has no money, and they have to buy books where to write, then you start writing on ten pages; does it make sense...” (KIIMO 2)*

### Strategies to improve patients’ clerkships

These were divided into measures to be taken by the health workers, those to be taken by the hospital leadership and those to be taken by the government.

#### Measures to be taken by health workers.

Holding each other accountable with respect to clerkship quality and completeness was suggested, including providing feedback from fellow health workers and from the records department.

*…like everyone I think should just be held accountable for their clerkship and give each other feedback…” (KIIMO 3)*

It was also suggested that medical students be mentored by senior doctors on the ward on the clerkship, and they should clerk the patients and present them to the senior doctors for guidance on the diagnosis and the management plan. This approach was believed to save time for senior doctors who may not have obtained time to collect information from patients and to facilitate the learning of students, most importantly ensuring the complete clerkship of patients.

*“…students can give us a very good clerkship if supervised well, then we can discuss issues of diagnosis, the investigations to be done and the management…” (KIIMO 1)*

Changes in the attitudes of health workers toward clerkships were suggested. This was also encouraged for those who work in laboratories to be able to perform the required investigations to guide diagnosis and management.

*“…our lab has the equipment, but they need to change their attitude toward doing the investigations…” (KIIMO 1)*

#### Measures to be taken by hospital leaders

The provision of tools to be used in clerkships was suggested as one of the measures that can be taken. Among the tools that were suggested include the following: a standardized clerkship guide, equipment for examination of the patients, such as blood pressure machines, and thermometers, among others. It was also suggested that a printer machine be used to print the clerkship guide to ensure the sustainability and availability of the tools. An electronic clerkship provision was suggested so that the amount of tedious paperwork could be reduced, especially for those who are comfortable with it.

*“…if the stakeholders, especially those who have funds, can help us to make sure that these tools are always available, it is a starting point…” (KIIOG 1)*

Continuous education of the clinicians about clerkships was suggested in the CMEs, and routine morning meetings were always held in the ward. Then, it was suggested that clinicians who clerked patients the best way are rewarded to motivate them.

*“…for the staff, we can may be continuously talking about it during our Monday morning meetings about how to clerk well and the importance of clerking…” (KIIOG 1)*

They also suggested providing a separate conducive room for the examination of patients to ensure the privacy of the patient, as this will ensure more detailed examination of the patients by the clinicians.

It was also suggested that more close supervision of the clerkship be performed and that a culture of good clerkship be developed to make clerkship a norm.

*“…as leaders of the ward and of the department, we should not get tired to talk about the importance of clerkship, not only in this hospital but also in the whole country…” (KIIOG 1)*

Proper record-keeping was also suggested, for people clerking to be assured that information will not be discarded shortly.

*“…because how good is it to make these notes yet we can’t keep them properly...” (KIIMO 2)*

It was also suggested that a records assistant be allocated to take notes for the clinicians to reduce their workload.

Coming up with SOPs, for example, putting different check points that ensure that a patient is fully clerked before the next step

*“…we can say, before a patient accesses theater or before a mother enters second stage room, they must be fully clerked, and there is a checklist at that point…” (KIIOG 1)*

Measures to be taken by the government

Improving the staffing level is strongly suggested to increase the health worker-to-patient ratio. This, they believed would reduce the workload off the health workers and allow them to give more time to the patients.

*“…we also need more staffing for the scan because the person who is there is overwhelmed…” (KIIMO 1)*

Staff motivation was encouraged through the enhancement of staff salaries and allowances. It was believed that it would be easy for these health workers to be supervised when they are motivated.

*“…employ more health workers, pay them well then you can supervise them well…” (KIIMO 1)*

Providing refresher courses to clinicians was also suggested so that they could be updated during the clerkship process.

Streamlining the referral system was also suggested through the use of lower health facilities so that some minor cases can be managed in those facilities to reduce the overcrowding of patients in the RRH.

*“…we need to also streamline the referral system, the way people come to the RRH; some of these cases can be handled in the lower health facilities; we need to see only patients who have been referred…” (KIIMO 2)*

**Fig. 8:**
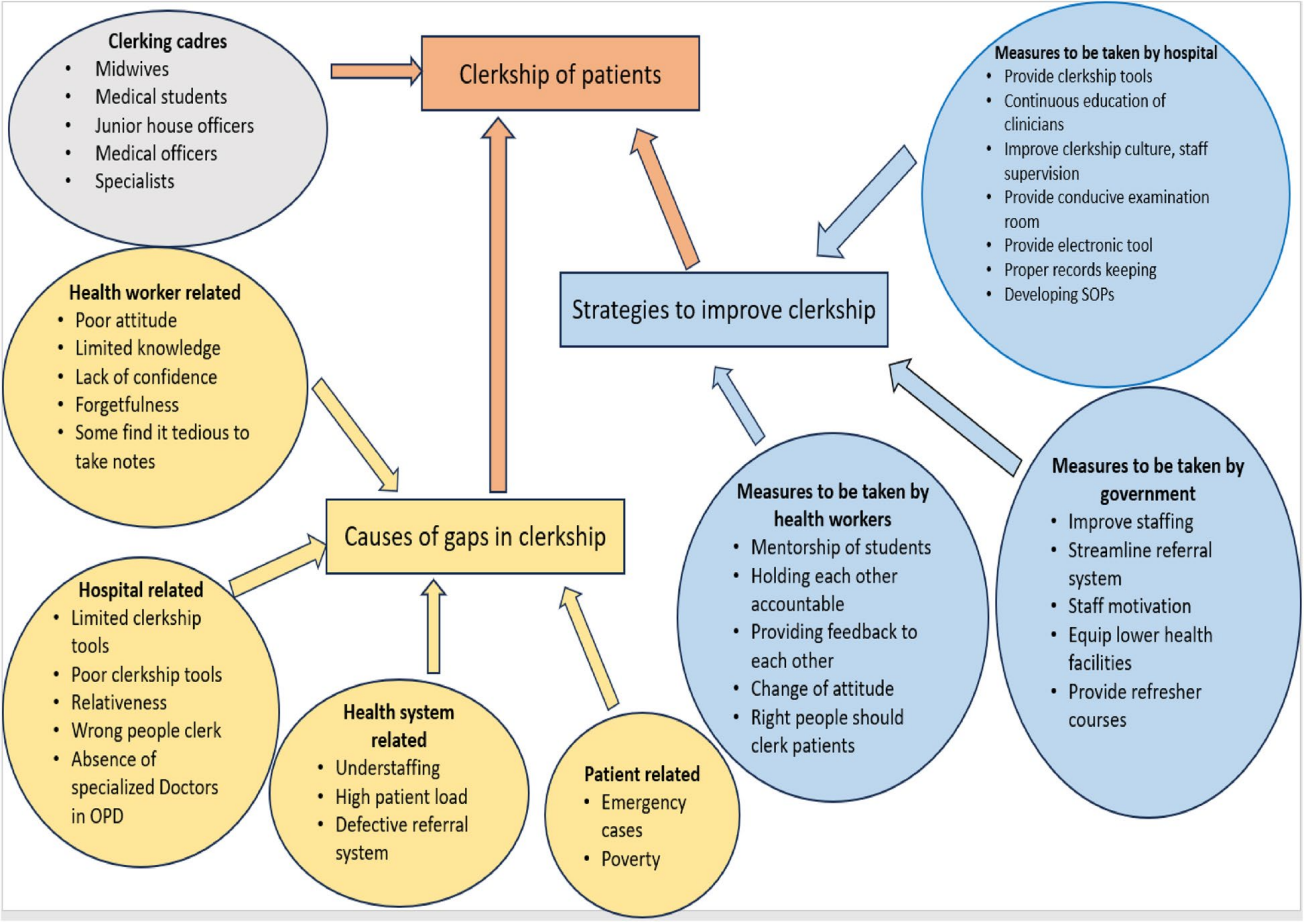
Scheme of the clerkship of patients, including the causes of the clerkship gap and the strategies to improve the clerkship at Mbale RRH

The qualitative results are further summarized in Fig. 8 below.

## Discussion of results

This study highlights a gap in the clerkships of patients admitted, treated, and discharged from the gynecological ward, with varying gaps in the different sections. This could be because some sections of the clerkship are considered more important than others. A study performed in Turkey revealed that physicians tended to record more information that aided their diagnostic tasks [11]. This is also reflected in the qualitative findings where participants expressed that particular information is required to make the diagnosis and not everything must be collected.

Biodata for patients were generally well recorded, and name and age were recorded for almost all the patients. A similar finding was found in the UK, where 100% of the patients had their personal details fully recorded [12]. Patient information should be carefully and thoroughly recorded because it enables health workers to create good rapport with patients and creates trust [13]. This information is also required for every interaction with the patient at the ward.

The presenting complaint, history of presenting complaint and the review of systems were fairly recorded, with each of them missing in less than 40% of the patients. The presence of a complaint is crucial in every interaction with the patient to the extent that a diagnosis can rarely be made without knowing the chief complaint [14, 15]. This applies to the history of presenting complaint as well [16]. For the 30% who did not have the presenting complaint recorded, this could mean that even the patient’s primary problem was not given adequate attention.

In the history, the greatest gap was noted in the history of current pregnancy, where many parameters were not recorded in most patients. This is, however, expected since the study was conducted on a gynecological ward, where only a few pregnant women are expected to visit, as they are supposed to go to their antenatal clinics [17]. However, there was also a large gap in past gynecological history, which is expected to be fully explored in the gynecology ward. A good medical history is key to obtaining a good diagnosis, in addition to a good clinical examination [3, 18]. Past obstetric history, past medical history, past surgical history, and family history also had large gaps, yet they are very important in the management of these patients.

The abdominal parameters, especially the pulse rate and blood pressure, were the least frequently recorded during the physical examination, and vital signs were most often recorded. However, there were substantial gaps in the general examination and vaginal examination. The least gap in vital sign examination is close monitoring, which is performed for most patients admitted to the ward due to the nature of the patients, some of whom are emergency patients [19].

Among the investigations, 29% of patients were not investigated. The least commonly performed investigations were pelvic USS and malaria tests, while complete blood count (CBC) was most commonly performed. Genital infections are among the most common reasons for women’s visits to health care facilities [20]. Therefore, most women in the gynecological ward are suspected to have genital tract infections, which could account for why CBC is most commonly performed.

The limited number of other investigations, such as pelvic ultrasound scans, underscore the relative contribution of medical history and physical examination to laboratory investigations and imaging studies aimed at making a diagnosis [1]. However, this would also highlight the system challenges of limited access to quality laboratory services in low- and middle-income countries [21]. This was also highlighted by one of the key informants who reported that the USS staff is available on some and not all days. This means that on days where the ultrasound department does not work, USS is not performed, even when needed.

We found that 20% of patients experienced prolonged hospitalization. This percentage is lower than the 24% reported in a study conducted in Ethiopia [22]. However, this study was conducted in a surgical ward. The median length of hospital stay was the same as that in a study conducted in Eastern Sudan among mothers following cesarean delivery [23]. A prolonged hospital stay has a negative impact not only on patients but also on the hospital [24, 25]. Therefore, health systems should aim to reduce the length of hospital stay for patients as much as possible to improve the effectiveness of health services.

At the multivariate level, abdominal examination was significantly associated with length of hospital stay, with patients whose abdominal examination was not complete being more likely to have a prolonged hospital stay. This underscores the importance of good examination in the development of proper management plans that improve the care of patients, hence reducing the number of days of hospital stay [5, 26].

## Conclusion

There is a gap in the clerkships of patients at the gynecological ward, which is recognized by the stakeholders at the ward. Some components of clerkships were recorded better than others, with the reasoning that clerkships should be targeted. There were no patients who received a complete clerkship. There was a significant association between clerkships and the length of hospital stay. The causes of the gap in clerkships were multifactorial and included those related to the hospital, those related to the health worker, those related to the health care system and those related to the patient. The strategies to improve the clerkship of patients also included measures taken by health care workers, measures taken by hospitals and measures taken by the government.

### Recommendations

Clerkship tools, such as the standardized clerkship guide and equipment for patient examination, were provided. The health workers were continuously educated on clerkships and trained on how to use the available tools. The development of SOPs for patient clerkships, the promotion of clerkship culture and the supervision of health workers.

### Strengths of the study

A mixed study, therefore, allows for the triangulation of results.

### Study limitations

The quantity of quantitative data collected, being secondary, is subject to bias due to documentation errors. We assessed the completeness of clerkship without considering the nature of patient admission. We did not record data on whether it was an emergency or stable case, which could be an important cofounder. However, this study gives a good insight into the status of clerkship in the gynecological ward and can lay foundation for future research into the subject.

### Supplementary Information

Supplementary Material 1. 

## Data Availability

The data and materials are available upon request from the corresponding author via the email provided.
