# Supplementary material for: Improving Clerkship to Enhance Patients’ Quality of care (ICEPACQ): a baseline study
Source: BMC Health Serv Res. 2024 Jul 26;24:852. doi: 10.1186/s12913-024-11337-w (PMC11282622; doi:10.1186/s12913-024-11337-w)
Supplement: Supplementary file 1 — Supplementary Material 1. [file 12913_2024_11337_MOESM1_ESM.docx]

**GAP IN CLERKSHIP OF PATIENTS ADMITTED, TREATED AND DISCHARGED FROM GYNECOLOGY WARD IN A REGIONAL REFERRAL HOSPITAL: A BASELINE STUDY**

**An interview guide**

1. **Do you think there is clerkship of patients on this ward? (***Opening question***)**
2. **Which people usually clerk patients on this ward?**
3. **How do you think is the quality and completeness of clerkship of the patients that are admitted and treated in this ward? *(****Get their opinion on how they see the quality and completeness of clerkship****)***
4. **Do you think clerkship of patients on this ward is done well? If not, why do you think there is a gap?** *(Probe about the 5 whys {Why....why…. why…. why…. why}).*
5. **What do you think can be done by the different stakeholders to improve the level of clerkship of the patients that are admitted and treated in this ward?** *(Probe about the following stakeholders: Health care workers, students the ward in charges and heads of departments, the hospital administration, the government).*
6. **What do you think must be provided in order to improve the clerkship of the patients? *(****Let them give the necessary requirements which they think must be provided in order to improve the clerkship****)***
